# Cellular senescence of renal tubular epithelial cells in renal fibrosis

**DOI:** 10.3389/fendo.2023.1085605

**Published:** 2023-02-28

**Authors:** Jun-Qing Zhang, Ying-Ying Li, Xue-Yan Zhang, Zeng-Hui Tian, Cheng Liu, Shi-Tao Wang, Fa-Rong Zhang

**Affiliations:** ^1^ College of Chinese Medicine, Shandong University of Traditional Chinese Medicine, Jinan, China; ^2^ College of First Clinical Medicine, Shandong University of Traditional Chinese Medicine, Jinan, China; ^3^ Department of Nephrology, Affiliated Hospital of Shandong University of Traditional Chinese Medicine, Jinan, China

**Keywords:** cellular senescence, renal fibrosis, renal tubular epithelial cells, oxidative stress, DNA damage, inflammation

## Abstract

Renal fibrosis (RF) is the common pathological manifestation of virtually all chronic kidney diseases (CKD) and one of the major causes of end-stage renal disease (ESRD), but the pathogenesis of which is still unclear. Renal tubulointerstitial lesions have been identified as a key pathological hallmark of RF pathology. Renal tubular epithelial cells are the resident cells of the tubulointerstitium and play an important role in kidney recovery versus renal fibrosis following injury. Studies in recent years have shown that senescence of renal tubular epithelial cells can accelerate the progression of renal fibrosis. Oxidative stress(OS), telomere attrition and DNA damage are the major causes of renal tubular epithelial cell senescence. Current interventions and therapeutic strategies for cellular senescence include calorie restriction and routine exercise, Klotho, senolytics, senostatics, and other related drugs. This paper provides an overview of the mechanisms and the key signaling pathways including Wnt/β-catenin/RAS, Nrf2/ARE and STAT-3/NF-κB pathway involved in renal tubular epithelial cell senescence in RF and therapies targeting renal tubular epithelial cell senescence future therapeutic potential for RF patients. These findings may offer promise for the further treatment of RF and CKD.

## 1 Introduction

CKD is a syndrome of persistent changes in the structure, function or both of the kidneys and affects the health of the individual. There has been an increase in CKD prevalence over the past few years, with a global prevalence of nearly 13% (1) and a prevalence of 10.8% in China (2). The high prevalence, disability and mortality rates of CKD, as well as the cost of health care, have made it a global public health concern, especially as the treatment for advanced CKD by renal replacement therapy including dialysis and kidney transplantation imposes a significant burden on patients (3). RF is a hallmark feature of kinds of kidney diseases and is the ultimate pathway for progressive kidney function, and improving RF can effectively delay the progression of CKD (4, 5). The main pathological manifestations of RF are glomerulosclerosis, fibrosis of the tubular interstitium and sclerosis or blockage of blood vessels in the kidney, however, the pathogenesis is not fully understood. Therefore, an in-depth investigation into the pathogenesis of CKD and RF and the search for safe and effective drugs for early intervention and treatment can not only solve the financial and mental burden for patients, but also save more medical resources and time costs for society. Cellular senescence occurs when cells are stressed to the point of irreversible cell cycle arrest. While senescent cells do not undergo cell cycle arrest, they remain high metabolic activity and secrete large quantities of cytokines affecting the surrounding microenvironment and neighboring cells cycle, called senescence-associated secretory phenotype (SASP). The morphology of senescent cells also differs from that of living cells; Researchers have observed *in vitro* cell cultures that senescent cells have larger and flattened cell bodies, vacuolated cytoplasm and abnormal organelles compared to healthy cells (6). Renal tubular epithelial cells are the resident cells of the renal tubular mesenchyme and are central to kidney recovery versus renal fibrosis following injury, which play a vital part in a number of acute and chronic kidney diseases (5, 7, 8). It has been shown that cellular senescence of renal tubular epithelial cells is a driving factor in the development of RF, and that delaying it is an effective measure to inhibit RF and an important strategy to slow down the progression of CKD (9, 10). There is a close relationship between renal tubular epithelial cells senescence and RF, and the onset of cellular senescence can accelerate this process, as this paper will illustrate.

## 2 Advances in cellular senescence-related mechanisms in renal tubular epithelial cells in RF

**Figure 1:**
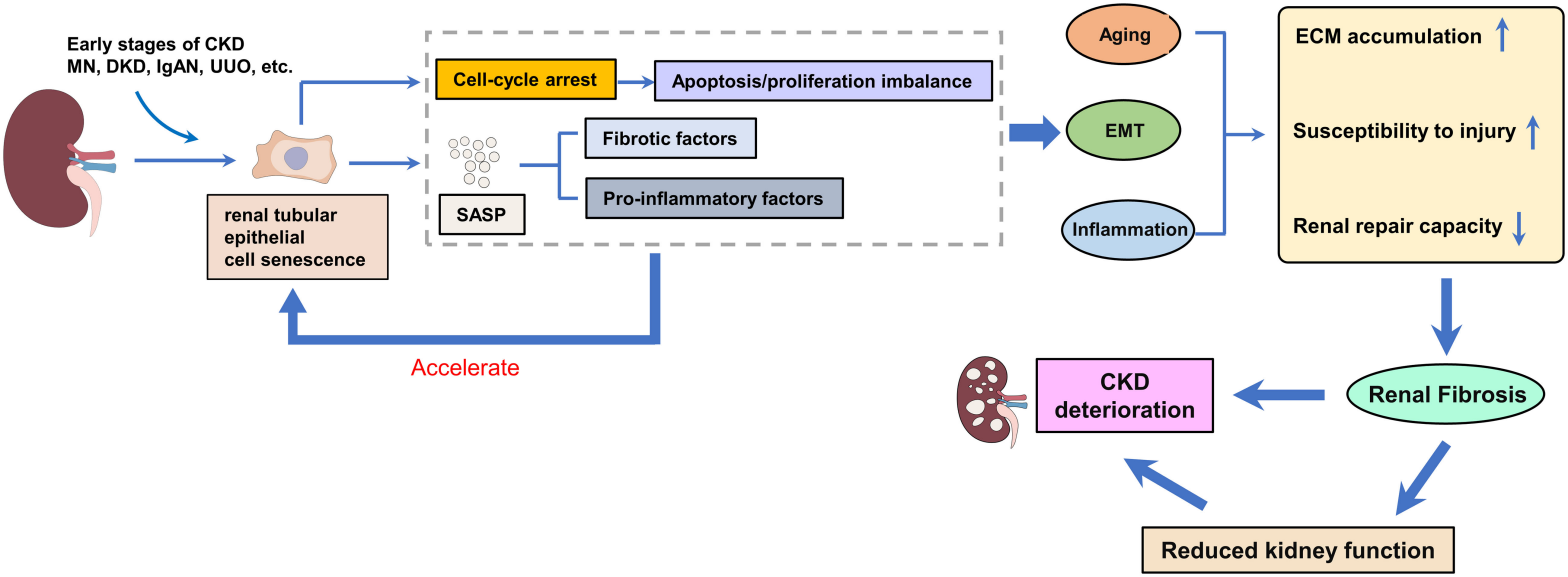
The effect of renal tubular epithelial cell senescence on renal fibrosis. Renal tubular epithelial cells are the main and earliest type of senescent cell to emerge. Senescent cells can secrete SASP (including pro-fibrotic and pro-inflammatory factors), which further accelerates the senescence of renal tubular epithelial cells, and the accelerated senescence of tubular epithelial cells drives the development of renal fibrosis.

Renal cell senescence was initially described in 1992 (11). Apart from its involvement in physiological renal ageing, senescence also plays an important part in the progression of CKD and acute kidney injury (AKI). It has been reported that accumulation of senescence markers and senescent cells can be detected in several experimental animal models such as ischemia-reperfusion (IRI) (12), unilateral ureteral ligation (UUO) (13) and in renal tissues of CKD patients (14–16). Clinical studies have also shown that accelerated senescent cells have been observed in the kidneys of patients with CKD in the early stages and even in the proteinuria phenotype with normal glomerular filtration rate (16). It is notable that these cells were mainly renal tubular epithelial cells (13), suggesting that renal disease develops and progresses in the presence of renal tubular epithelial cells senescence. In addition, researchers have found that cellular senescence leads to kidney dysfunction and accelerates the progression of kidney disease (16, 17). In CKD, as cellular senescence occurs, senescent cells lose their ability to grow and repair while secreting SASP components (10, 18), including pro-inflammatory factors such as interleukin-1 (IL-1) and interleukin-6 (IL-6), and pro-fibrotic mediators such as transforming growth factor-β1 (TGF-β1) and matrix metalloproteinases (MMPs). These SASP factors, on the one hand, promote the cell’s own senescence and senesce neighboring cells in a paracrine manner. On the other hand, pro-inflammatory factors can lead to an inflammatory response and promote the phenotypic transformation of intrinsic cells, causing them to release a series of toxic factors. These toxic factors, together with the pro-fibrogenic mediators secreted by senescent cells, can contribute to Epithelial-mesenchymal transition(EMT) as well as the proliferation and differentiation of renal interstitial fibroblasts and their transformation into myofibroblasts, resulting in an increase in matrix protein synthesis and a decrease in degradation, and a large amount of extracellular matrix deposition in the renal parenchyma, destroying the normal structure of renal tissue, promoting the formation of fibrous scar, which can facilitate the progression of RF and induce irreversible structural damage to the kidney and renal hypofunction or even loss, ultimately leading to renal failure (6, 19). Moreover, the regenerative capacity of aging kidney cells is significantly reduced, contributing to maladaptive renal repair, which further expedites kidney aging and CKD progression, ultimately accounting for RF (20). Consequently, the accelerated senescence of renal tubular epithelial cells is a driving factor in the development of RF (**Figure 1**), and it is essential to investigate the pathogenesis of ageing in renal tubular epithelial cells and find the regulators that target the senescence of tubular epithelial cells to prevent and control the progression of RF, which can provide valuable therapeutic targets for treating CKD. This section reviews the mechanisms of renal tubular epithelial cell senescence in RF.

### 2.1 Oxidative stress

**Figure 2:**
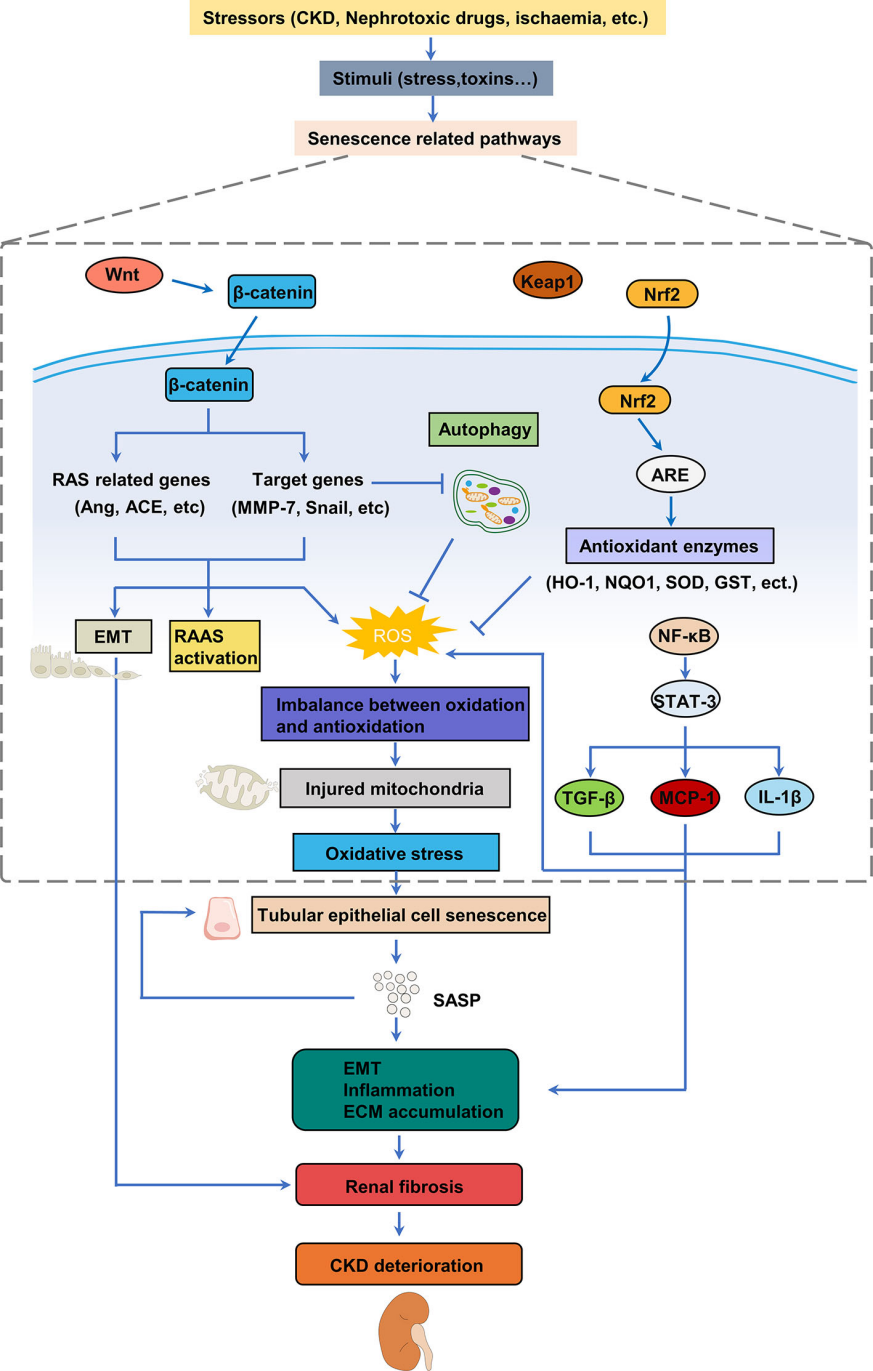
Mechanisms involved in oxidative stress of cellular senescence in renal fibrosis. Various stimulus can trigger oxidative stress by activating different pathways to produce ROS in the kidney, thereby leading to the senescence of renal tubular epithelial cells. Senescence of tubular epithelial cells promotes EMT and leads to the SASP, which increases inflammation and pathological accumulation of extracellular matrix, ultimately resulting in fibrosis and the development of CKD. CKD, chronic kidney disease; EMT, epithelial-mesenchymal transition; SASP, senescence-associated secretory phenotype; ECM, extracellular matrix; ROS, reactive oxygen species; TGF-β, transforming growth factor β; MCP-1, monocyte chemoattractant protein-1.

OS is the driving force responsible for the loss of renal tubules, glomeruli and endothelial cells, which plays an essential part in the onset and progression of CKD and aging (21–23). OS occurs when oxidant compounds are generated in excess of antioxidant defense mechanisms as a result of external or internal irritants, creating an imbalance, favoring oxidation, resulting in the infiltration of neutrophils, the secretion of proteases, and the generation of large volumes of oxidative intermediates (24). OS is caused by free radicals and is thought to be a contributing factor to cell senescence and CKD (25). An extensive range of acute and chronic kidney diseases involve mitochondrial dysfunction first and foremost in tubular cells (26–28). Reactive oxygen species (ROS) are produced by mitochondria when their morphology changes and their function is lost which will lead to OS and inflammation, triggering DNA damage (24, 29). OS can cause accelerated telomere attrition and cellular senescence, and further exacerbates the progression of RF (30). In this review, we have focused on the crucial signaling pathways of OS, as below and in **Figure 2**.

#### 2.1.1 Related signaling pathways

##### 2.1.1.1 Wnt/β-catenin/RAS

It is found that Wnt/β-catenin/RAS signaling is essential to mediate cellular senescence-driven RF and implicated in mitochondrial dysfunction (27). Wnt/β-catenin signaling is involved in organ development and tissue repair and is an evolutionarily highly conserved signaling pathway which is silent in normal adult kidneys (31). It is reactivated in different types of CKD by aberrant activation following renal injury and plays a key role in subsequent repair or disease progression following multiple injuries and its abnormal expression is highly correlated with tubulointerstitial fibrosis (12, 32, 33). Wnt/β‐catenin signaling is a notable regulatory element of RAS. The Wnt signaling pathway is activated in the pathological state, causing the disruption of the downstream β-catenin pathway of degradation and resulting in a massive upregulation of its expression. As β-catenin accumulates in large amounts, it undergoes nuclear translocation and enters the nucleus to activate downstream target genes highly relevant to renal fibrosis (fibronectin, Snail1, MMP-7, etc.) and various RAS genes (e.g. Ang, ACE, AT1R), thereby promoting cell proliferation, RAAS activation, and the occurrence of EMT, contributing to the pathological accumulation of extracellular matrix, which can lead to the initiation of RF (32, 34, 35). Consequently, repression of Wnt/β-catenin enables multiple RAS genes to be blocked simultaneously. The findings indicate that Wnt/β‐catenin/RAS signaling pathway plays a crucial role in mitochondrial dysfunction, which is likely to cause the overproduction of ROS in the body, triggering the OS and DNA damage, eventually leading to cellular aging and RF (27). Repression of Wnt/β-catenin can slow down the progression of RF by better blocking RAS and protecting mitochondrial function.

##### 2.1.1.2 Nrf2/ARE

The Nuclear factor erythroid 2-related factor 2 (Nrf2)/antioxidant response element (ARE) signaling pathway is one of the most essential antioxidant regulatory pathways, playing pivotal parts in the mediation of the acclimated stress response of cells to oxidation (36). Nrf2, a redox-sensitive transcription factor encoded by the gene NFE2L2, is a primary regulator of antioxidant enzymes that protect the body from OS and inflammation. Heme oxygenase-1 (HO-1), one of the targets of Nrf2-ARE, is an essential antioxidant enzyme, which can be involved in antioxidant activities in cells and tissues and provides a protective shield against renal injury (37). NAD(P)H: quinone oxidoreductase 1 (NQO1) can suppress the reductive activity of electrons and decrease the formation of ROS through its own enzymatic effect, thus increasing the level of NQO1 is associated with a reduced susceptibility to oxidative damage. Under normal physiological conditions, Nrf2 is anchored in the cytoplasm to the proteasomal degradation by combining with Kelch-like ECH-associated protein 1 (Keap1), while Keap1 is regenerated (38). In response to oxidative damage, Nrf2 separates from Keap1 and free Nrf2 accumulates in the cytoplasm before translocating to the nucleus where it binds to the ARE and activates the transcription of downstream antioxidative enzymes, such as HO-1, NQO1, superoxide dismutase (SOD), glutathionine S-transferase (GST), glutathione reductase (GR) and glutamate-cysteine ligase catalytic subunit (GCLC), to modulate cell redox balance and reduce the extent of damage (39–41). Recent researches have revealed that activation of a variety of signaling pathways, for instance phosphatidylinositide 3-kinases (PI3K)/protein kinase B (Akt) and mitogen-activated protein kinase (MAPK), could promote the liberation of Nrf2 from Keap1 as well as its subsequent translocation to induce the downstream expression of diverse antioxidant proteases (42, 43). Downregulation of Nrf2/ARE can accelerate cellular senescence by promoting oxidative stress, which suggests that drugs targeting Nrf2 signaling can inhibit cellular senescence. In addition, studies demonstrated that the NRF2 pathway may be a critical connection between OS and ferroptosis. The reason why ferroptosis can be attenuated by suppressing mitochondrial oxidative stress through modulation of the Nrf2/ARE pathway is that it represses the capability of Nrf2 to bind ARE within GPX4 and SLC7A11 gene promoters, which in turn induces transcriptional silencing (44). Ferroptosis is characterized by intracellular iron accumulation and lipid peroxidation during cell death, which has been shown the potential association with CKD (45, 46). The connection between Nrf2/ARE and kidney diseases has been described in various reports (25, 37, 47). The lack of Nrf2 can accelerate renal injury in various models, while enhancing Nrf2 activity in renal tubules can dramatically reduce the damage associated with AKI and prevent AKI from progressing to CKD by reducing oxidative stress (48). On the other hand, activation of Nrf2 also protects against fibrosis. In mouse models of unilateral ureteral obstruction, it was observed that Keap1 was downregulated, allowing activation of Nrf2 pathway thereby preventing the production of ROS and improving fibrosis. Nevertheless, prolonged obstruction results in a gradual decrease in nuclear Nrf2, as well as a reduction in antioxidant levels, which leads to increased oxidative stress, inflammation, fibrosis and tubular damage (25). In conclusion, targeted modulation of Nrf2/ARE can ameliorate oxidative damage, in consequence delaying the aging of renal tubular epithelial cells and inhibiting the process of fibrosis and CKD.

##### 2.1.1.3 STAT-3/NF-κB

The activation of signal transducer and activator of transcription-3 (STAT-3)/activated nuclear factor-κB (NF-κB) signaling pathway has been suggested to be regulatory mechanisms of inflammatory factors including TNFα and IL-6 and associated with the progression of CKD. It was reported that RF reduces renal antioxidant enzyme activation, while oxidative injury also plays a major role in fibrosis. After kidney injury, ROS production increases the expression of NF-κB and pro-inflammatory factors (49, 50) and activates the STAT-3 pathway (51), while NF-κB and pro-inflammatory cytokines further induce the production of excessive ROS (52). These processes lead to the disruption of cellular redox homeostasis and growth of mitochondrial damage, and stimulate the recruitment of inflammatory cells and infiltration of interstitial inflammatory macrophages, and directly augment myofibroblast activation through increasing the production of TGF-β1, IL-1β, and MCP-1 (53), causing OS and inflammation (54), leading to further damage to tubular cells and ultimately to the senescence of renal tubular epithelial cells and RF (55).

#### 2.1.2 Related molecules

MicroRNAs (miRNAs) were first described in Caenorhabditis elegans1 (56) and are highly conserved among species. MiRNAs regulate gene expression following transcription and govern bioprocesses. MiRNAs are integral not only to growth and homeostasis, but also have a significant impact on pathophysiology in the kidney (57, 58). A number of miRNAs have been proven to either kick-start or sustain the cellular reaction that results in fibrosis (59). Studies demonstrated that miR-21 could contribute to renal fibrosis by targeting Smad7, which is a negative regulator of TGF-β1/Smad3 signaling, inducing metabolic disturbances (58). It is reported that miR-9-5P can prevent the down-regulation of genes linked to crucial pathways of metabolism, in particular mitochondrial function, oxidative phosphorylation (OXPHOS), fatty acid oxidation (FAO) and glycolysis, impede TGF-β1-induced bioenergetic disorders, reduce the expression of pro-fibrotic markers in proximal renal tubule cells, and act to delay tubular epithelial cells senescence and inhibit RF (60). In addition, overexpression of miR-214 induces apoptosis and disrupts mitochondrial oxidative phosphorylation in response to a variety of injuries. The proximal tubule-specific absence of MiR-214 impairs inflammation, apoptosis, fibrosis and mitochondrial damage (61). Such findings have highlighted the potential of miRNA as a therapeutic candidate and diagnostic biomarker for renal tubular epithelial cells senescence and RF.

### 2.2 Telomeres doctrine

Telomeres are nuclear protein structures located at the ends of eukaryotic chromosomes that maintain the integrity of the genome where telomeres prevent DNA damage and provide a key function in the manipulation of cellular senescence and physical ageing (62). The dysfunction of telomeres results from exceedingly short telomeres or altered telomere structure, and the progressive loss of telomeres contributes to the exposure of DNA ends, causing activation of DNA damage responses (DDRs) (63), ultimately culminating in replicative cellular senescence and chromosomal instability, both of which are significant markers of senescence (64). Investigations support that telomere attrition may have a causal effect on the risk of CKD and that decompensated renal function may be causative for accelerating telomere attrition (65).

Disruptor of telomeric silencing 1-like (Dot1L) is situated on the nucleosome surface (66) which is associated with a number of biological processes, for instance the regulation of transcription, DDRs, cell cycle progression and embryonic cell development (67, 68). It has been found that inhibition of Dot1L decreases ROS levels, attenuates OS and inhibits RF by reducing PI3K/AKT signaling (69). In summary, these results have demonstrated that Dot1L inhibitors could be a prospective target for treatment in RF

### 2.3 DNA damages

DNA damage is a hallmark of numerous forms of nephron damage, activating a series of cell signaling cascades called DDRs to undergo the restoration of DNA integrity, cell cycle arrest in G2/M, cell senescence and cell death, which will cause the onset and progression of RF (70, 71). DNA double-strand breaks (DSBs) are the most powerful initiators of DDRs, and p53/p21 as well as p16/p16INK4a-pRB signal pathway are the two major modulators of these responses (72). It is suggested that targeting DNA damage and repair might serve as an attractive tactic to safeguard the kidney in RF and CKD. Cellular communication network factor 2 (CCN2) was detected to exacerbate DNA damage and the consequent DDR–cellular senescence–fibrosis sequence after kidney lesions (73, 74). Multiple studies have shown that fatty acids (FA) especially saturated FA are likely to be responsible for the induction of the NLRP3 inflammasome *via* generating ROS, and more elevated levels of ROS can account for oxidative DNA damage (75, 76). It is beneficial to protect the renal tubular epithelial cells from DNA damage by mediating fatty acid uptake and CCN2 inhibition, thereby inhibiting cellular senescence and the progression of RF. Nicotinamide mononucleotide (NMN) has been demonstrated to display beneficial impacts on attenuating RF by repressing tubular DNA damage and cellular aging (77), and NMN administration may be a useful avenue for the prevention or treatment of RF (78).

### 2.4 Strategies to improve senescence of renal tubular epithelial cells

Renal tubular epithelial cell senescence plays an essential role in the progression of renal fibrosis and CKD. Therefore, strategies to improve senescence of renal tubular epithelial cells, known as senescence therapies, are also potential therapies for these diseases (6). The available interventions for senescence include non-pharmacological therapies (such as caloric restriction and routine exercise), Klotho, drugs that selectively eliminate senescent cells (known as senolytics), drugs that inhibit SASP (known as senostatics) and other related drugs (**Table 1**).

**Table 1:** Senotherapeutic approaches.

Senotherapeutic approach	Type	Examples	Refs.
Non-pharmacological therapies	Lifestyle interventions	Caloric Restriction	(79, 80)
		Routine Exercise	(81–84)
Therapeutic Potential	Increase of anti-aging protein Klotho expression	angiotensin II receptor antagonists, PPAR-γ agonists, Paricalcitol and statins	(85–87)
Senolytics	selectively remove senescent cells	D+Q group, ABT⁃263, FOXO4⁃DRI	(88–91)
Senostatics	SASP regulators	rapamycin, metformin, and resveratrol	(92–96)
Other Drugs	Inflammation inhabitation	NR, NMN, NSAIDs	(77, 97, 98)

SASP, senescence-associated secretory phenotype; D+Q group, dasatinib and quercetin.

#### 2.4.1 Caloric restriction

Caloric restriction (CR), which refers to limiting caloric intake without causing damage to the body, is recognized as an effective strategy for maintaining health and prolonging life. CR reduces the expression of aging markers in kidney, and can also delay cellular senescence and changes associated with cellular senescence within kidney, for example glomerulosclerosis, tubular atrophy and interstitial fibrosis (79). The underlying mechanisms involve inhibition of insulin-like growth factor-1 (IGF-1) activation, triggering autophagy through modulating primary metabolic signaling pathways, such as inhibition of mTOR and activation of SIRT1, AMPK, which can reduce oxidative damage (80, 99). Recently, it was found that endogenous hydrogen sulfide (H2S) mediated amino acid restriction, particularly methionine restriction, and exerted a positive effect in inhibiting the protein effect of SASP production in aged kidneys (100, 101). In addition to its antioxidant capacity, H2S also significantly reduces inflammatory cell infiltration, decreases pro-inflammatory cytokines such as TNF-α, IL-6, which inhibits progression of tubular epithelial cells senescence and RF (102–104).

#### 2.4.2 Routine exercise

Routine exercise is a promising lifestyle intervention to slow down ageing extend life span. The exercise intervention could result in participants showing measurable declines in BMI, waist circumference and fat mass, as well as a marked decrease in biomarkers of cellular ageing (81). There has been evidence that regular exercise can reduce both ROS and serum AGE levels, attenuating the oxidative stress caused by ageing (82). Interestingly, new study reveals that exercise training can enhance telomerase reverse transcriptase gene expression and telomerase activity, attenuating attrition of telomere and thereby slowing cellular ageing (83). Moreover, another study shows that the combination of melatonin and exercise attenuated metabolic syndrome and reduced anxiety and depression of type 2 diabetic rats by modulating insulin resistance, inflammatory cytokines, mitochondrial biogenesis and ATP levels (84). On the other hand, a meta-analysis reveals that exercise is beneficial in improve the blood pressure profile and significantly reduced VO2 levels of patients with renal failure (105). A latest study also demonstrates that treadmill exercise seems to be a useful intervention for inducing improved peripheral circulation. And it suggests that treadmill exercise should be performed under or near an ambient temperature of 20°C (106). Therefore, it is necessary to take long-term and regular exercise to maintain health and slow down the aging process.

#### 2.4.3 Therapeutic potential of Klotho

The anti-aging protein Klotho, encoded by the anti-aging gene Klotho, is a single-channel transmembrane protein, mainly α-Klotho protein, divided into membrane and soluble forms. Klotho is highly expressed in normal kidneys, especially in the distal tubules, but oxidative stress, inflammation, angiotensin II, aldosterone and proteinuria, which are present during organ and organism aging and various CKD pathologies, can reduce Klotho expression (107). Klotho is a pleiotropic protein that down-regulates a variety of cytokines and growth factors, such as IGF-1, Wnt/β-catenin and TGF-β1, and plays a variety of biological functions such as regulating cellular senescence, inhibiting apoptosis, inflammation, oxidative stress and regulating calcium and phosphorus metabolism (12, 108, 109), which plays a critical role in delaying renal aging, protecting the kidney from acute and chronic injury, promoting restoration of renal function and delaying the progression of RF and CKD (85, 86, 110). Therefore, it is possible to interfere with renal senescence by upregulating Klotho expression to inhibit RF and delay CKD progression. Researches has showed that Studies have shown that many drugs such as angiotensin II receptor antagonists, PPAR-γ agonists, Paricalcitol and statins may increase endogenous Klotho expression (86, 87). However, to date, there is insufficient clinical evidence for the effects of these drugs on Klotho.

#### 2.4.4 Senolytics

Senolytics are a class of drugs that selectively remove senescent cells, including dasatinib and quercetin (D+Q group), ABT⁃263, FOXO4⁃DRI, etc. Recent studie has found that the D+Q group can reduce the expression of senescence marker proteins such as SA⁃β⁃gal, p16, p21 and inflammatory factors such as IL⁃6 and MCP⁃1 in mouse renal tubular epithelial cells, suggesting that Senolytics could inhibit the progression of RF by delaying the senescence of renal tubular epithelial cells (9, 88). ABT ⁃263 can inhibit the activity of the anti-apoptotic proteins B lymphocytoma 2 (BCL ⁃2) and BCL ⁃XL to induce apoptosis in senescent cells and exerts an anti-aging effect (89, 90). FOXO4 ⁃DRI, a peptide that interferes with the function of FOXO4 protein, competitively inhibits the binding of FOXO4 to p53 in senescent cells and induces apoptosis to delay tubular senescence and renal hypofunction (91). These discoveries unlock an emerging and encouraging therapeutic route for the treatment of CKD and its co-morbidities through optionally targeting senescent cells.

#### 2.4.5 Senostatics

Senostatics are drugs that inhibit the aging phenotype and reduce SASP secretion while maintaining cell viability (18),including rapamycin, metformin, and resveratrol. These compounds can activate autophagy, improve mitochondrial function and inhibit IL-6, IL-8 and other SASP expression, reduce OS (92–95), decrease SA-β-gal levels in tubular epithelial cells (96), thereby delaying the aging of renal tubular epithelial cells and inhibiting RF.

#### 2.4.6 Other drugs

Other candidate drugs include NR and NMN that are NAD+ precursor supplements, and non-steroidal anti-inflammatory drugs (NSAIDs), which can inhibit proinflammatory signaling pathways to reduce inflammation and finally exert an anti-ageing effect (77, 97, 98).

## 3 Conclusion

**Figure 3:**
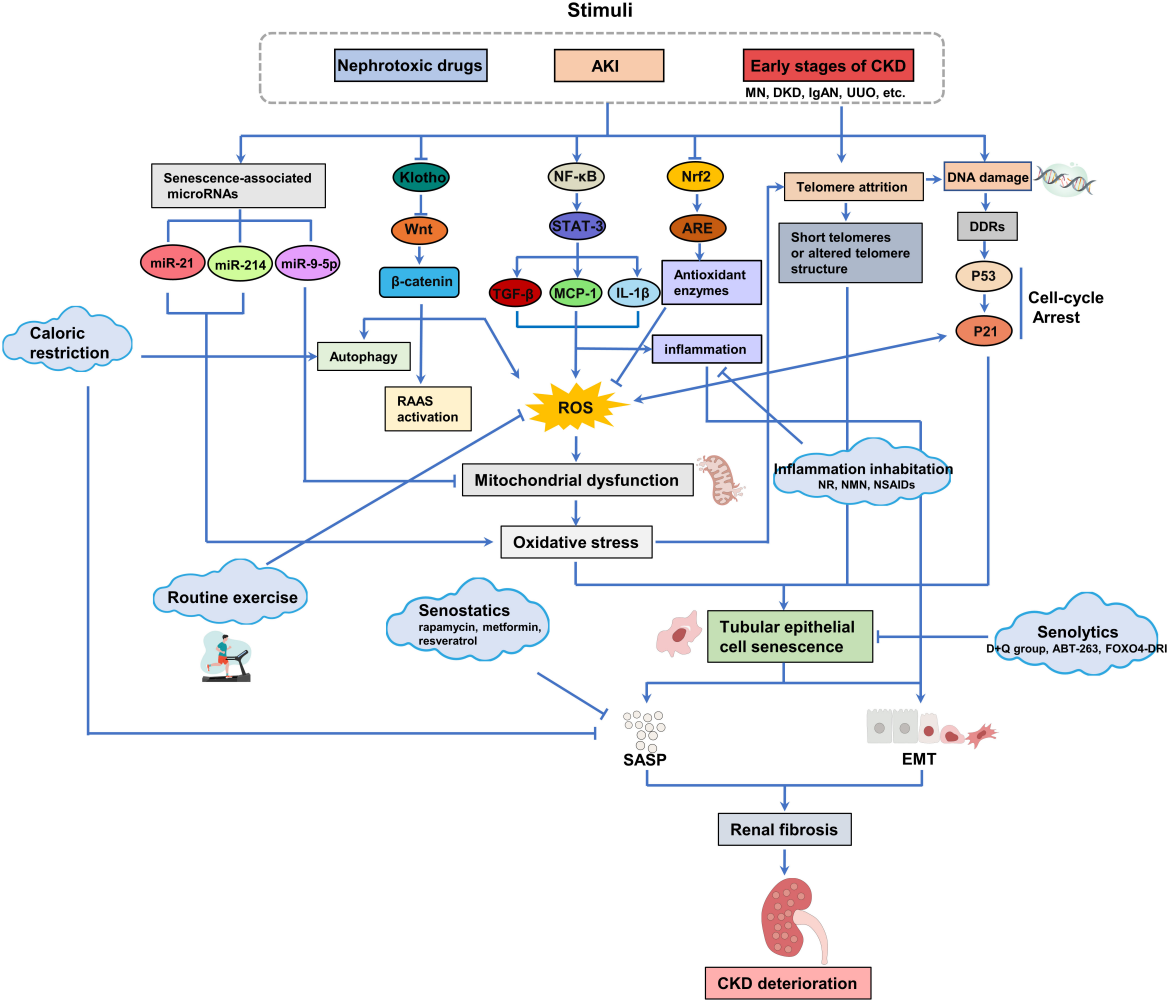
Oxidative stress, telomere attrition and DNA damage caused by stress, chemical or reactive oxygen species (ROS) accumulation are the main reasons for the senescence of renal tubular epithelial cells. The presence of senescent tubular epithelial cells in the kidney can be detected in the early stages of CKD. Senescent cells secrete SASP (including pro-fibrotic and pro-inflammatory factors), which further accelerates the senescence of tubular epithelial cells and drives the progression of RF. Available interventions for senescence include calorie restriction and regular exercise, Klotho, senolytics, senostatics and other related drugs, which offer promise for the treatment of renal fibrosis and CKD associated with renal tubular epithelial cell senescence.

Renal tubular epithelial cells senescence is a driver of RF, and the pathogenesis associated with it includes oxidative stress, telomere shortening, and DNA damage. These factors interact and synergistically contribute to the development of cellular senescence in RTECs. In-depth studies on the molecular mechanisms of RF associated with senescence in renal tubular epithelial cells are expected to provide potentially viable therapeutic ideas for RF, such as caloric restriction, routine exercise, endogenous Klotho, Senolytics, Senostatics, NAD+ supplementation, NSAIDs (**Figure 3**).

Although the nephroprotective effects of the above therapies are hopeful, and many have been studied in animal models and *in vitro*, clinical trials of targeted therapeutic strategies are required to assess their safety and efficacy in patients. Moreover, many inflammatory and fibrotic factors are also SASP components and their expression is not adequate to recognize cellular senescence. Given that the available senescence indices may vary among conditions and organs, there is a need for highly sensitive and specific non-invasive methods of detecting senescence (6).In future studies, it would be beneficial to search for more therapeutic targets for RF-related cellular senescence to further facilitate the development and screening of relevant drugs, which will provide new points for the prevention and treatment of CKD and RF.

## Author contributions

J-QZ and Y-YL decided on the topics. X-YZ and CL and S-TW reviewed the literature. J-QZ and Z-HT prepared the figures and table. J-QZ wrote the manuscript. F-RZ revised the manuscript. All authors contributed to the article and approved the submitted version.
